# A gene on the *HER2* amplicon, C35, is an oncogene in breast cancer whose actions are prevented by inhibition of Syk

**DOI:** 10.1038/sj.bjc.6605763

**Published:** 2010-07-13

**Authors:** E Katz, S Dubois-Marshall, A H Sims, D Faratian, J Li, E S Smith, J A Quinn, M Edward, R R Meehan, E E Evans, S P Langdon, D J Harrison

**Affiliations:** 1 Breakthrough Research Unit and Division of Pathology, Institute of Genetics and Molecular Medicine, University of Edinburgh, Crewe Road, Edinburgh EH4 2XU, UK; 2 Vaccinex Inc., 1895 Mt Hope Avenue, Rochester, NY, USA; 3 Section of Dermatology, Division of Cancer Sciences, Faculty of Medicine, University of Glasgow, Glasgow, UK; 4 MRC Human Genetics Unit, Institute of Genetics and Molecular Medicine, Western General Hospital, Edinburgh EH4 2XU, UK

**Keywords:** breast cancer, C35, HER2, epithelial to mesenchymal transition, ITAM, Syk kinase

## Abstract

**Background:**

C35 is a 12 kDa membrane-anchored protein endogenously over-expressed in many invasive breast cancers. C35 (*C17orf37*) is located on the *HER2* amplicon, between *HER2* and *GRB7*. The function of over-expressed C35 in invasive breast cancer is unknown.

**Methods:**

Tissue microarrays containing 122 primary human breast cancer specimens were used to examine the association of C35 with HER2 expression. Cell lines over-expressing C35 were generated and tested for evidence of cell transformation *in vitro*.

**Results:**

In primary breast cancers high levels of C35 mRNA expression were associated with *HER2* gene amplification. High levels of C35 protein expression were associated with hallmarks of transformation, such as, colony growth in soft agar, invasion into collagen matrix and formation of large acinar structures in three-dimensional (3D) cell cultures. The transformed phenotype was also associated with characteristics of epithelial to mesenchymal transition, such as adoption of spindle cell morphology and down-regulation of epithelial markers, such as E-cadherin and keratin-8. Furthermore, C35-induced transformation in 3D cell cultures was dependent on Syk kinase, a downstream mediator of signalling from the immunoreceptor tyrosine-based activation motif, which is present in C35.

**Conclusion:**

C35 functions as an oncogene in breast cancer cell lines. Drug targeting of C35 or Syk kinase might be helpful in treating a subset of patients with *HER2*-amplified breast cancers.

The gene C35 (*C17orf37*) is located within the smallest region of amplification of the *HER2* amplicon, between *HER2* and *GRB7*. It is a 12 kDa membrane-anchored protein over-expressed in 40–50% of invasive breast cancers (Evans *et al*, 2006). C35 has recently been implicated in conferring invasive potential in prostate cancer cell lines (Dasgupta *et al*, 2009). It contains a canonical immunoreceptor tyrosine-based activation motif (ITAM; Evans *et al*, 2006), a motif common in receptors of the immune system (Underhill and Goodridge, 2007), which has been associated with cell transformation through the activation of downstream Syk signalling. This raises the possibility that C35 can function as a transforming oncogene. The ability of ITAM-containing proteins to transform non-haematopoietic cells was previously shown using viral glycoproteins, such as the murine mammary tumour virus envelope protein (MMTV Env; Katz *et al*, 2005). Other examples of non-haematopoietic transformation by ITAM-containing proteins include latent membrane protein 2A of Epstein–Barr virus in skin keratinocytes (Lu *et al*, 2006) and K1 protein of Kaposi's sarcoma-associated herpes virus in endothelial cells (Wang *et al*, 2006). Particularly relevant were the observations that ITAM-containing proteins contribute to mammary epithelial cell (MEC) transformation and development of mammary carcinomas (Katz *et al*, 2005; Grande *et al*, 2006; Ross *et al*, 2006).

Using the ITAM-containing envelope protein of MMTV Env and a chimeric B-cell receptor protein, many researchers have made several key observations (Katz *et al*, 2005; Grande *et al*, 2006; Ross *et al*, 2006): (1) ITAM-containing protein expression can transform immortalised normal MECs in three-dimensional (3D) culture; (2) ITAM-induced transformation is dependent on its tyrosine phosphorylation and is associated with downstream Src and Syk kinase activation and (3) mutation of the ITAM tyrosines reduces tumour induction markedly by MMTV *in vivo* and influences its genomic integration. Therefore, ITAM-containing protein expression can switch on an intrinsic transformation programme in MECs. This programme is closely associated with epithelial to mesenchymal transition (EMT). Whereas epithelial markers such as E-cadherin and keratin-18 are down-regulated, mesenchymal markers such as N-cadherin and vimentin are up-regulated (Katz *et al*, 2005; Grande *et al*, 2006).

In this study, we determined the co-expression of C35 and HER2 proteins in human breast cancers. High levels of C35 expression were shown to induce invasion mediated by EMT *in vitro* 3D cultures using cell lines. Mutation of ITAM of C35 (or downstream Syk inhibition) was sufficient for the reversal of C35-induced transformation. Syk inhibition in combination with anti-HER2 therapy was shown to be effective in BT474 cell line model, offering a possible therapeutic approach to treat HER2^+^ tumours.

## Materials and methods

### Tissue microarray construction and AQUA analysis

The population characteristics of the trastuzumab-treated cohort are summarised in Supplementary Table S1. *HER2* gene amplification status was confirmed by fluorescence *in situ* hybridisation (FISH) according to the manufacturer's recommendations (*HER2* FISH PharmDx; Dako, Ely, Cambridge, UK). The use of this cohort was approved by the Lothian Research Ethics Committee (08/S1101/41). After H&E sectioning of representative tumour blocks, tumour areas were marked for TMA construction and 0.6 mm^2^ cores were placed into three separate TMA replicates for each sample, as previously described (Kononen *et al*, 1998).

Immunofluorescence was carried out using methods previously described (Camp *et al*, 2002). Pan-cytokeratin antibody was used to identify infiltrating tumour cells and normal epithelial cells, DAPI counterstain to identify nuclei and Cy-5-tyramide detection for target (C35, 1 : 500 dilution; Vaccinex, Rochester, NY, USA) for compartmentalised (tissue and subcellular) analysis of tissue sections. Monochromatic images of each TMA core were captured at × 20 objective using an Olympus AX-51 epifluorescence microscope (Olympus, Southend-on-Sea, UK), and high-resolution digital images analysed by the AQUAnalysis software (HistoRx Ltd., Branford, CT, USA). Briefly, a binary epithelial mask was created from the cytokeratin image of each TMA core. Similar binary masks were created for cytoplasmic and nuclear compartments on the basis of DAPI staining of nuclei. C35 expression was quantified by calculating the Cy5 fluorescent signal intensity on a scale of 0–255 within each image pixel, and the AQUA score was computed by dividing the sum of Cy5 signal within the epithelial mask by the area of the cytoplasmic compartment.

AQUA scores were averaged from replicate cores. If the tumour epithelium comprised <5% of total core area, the core was excluded from analysis. To determine the cut-point value for C35 expression in Kaplan–Meier analysis (Altman *et al*, 1994), we used X-Tile (Yale University New Haven, CT, USA), which allows determination of an optimal cut point while correcting for the use of minimum *P* statistic (Camp *et al*, 2004). Overall survival was subsequently assessed by Kaplan–Meier analysis with log-rank for determining statistical significance. Comparison of differences in means of C35 according to HER2 status was carried out using the Student's *t*-test. All calculations and analyses were two tailed where appropriate using SPSS 14.0 for Windows (SPSS Inc., Chicago, IL, USA).

### Immunohistochemistry

The following antibodies were used: C35, an affinity-purified rabbit polyclonal antibody 78.2 (Vaccinex) at 0.42 *μ*g ml^−1^; cytokeratins 5/6 (CK5/6), rabbit polyclonal antibody (Dako) at 1 : 50 dilution; E-cadherin, mouse monoclonal (BD Biosciences, Oxford, UK) at 1 : 450 dilution; Twist, mouse monoclonal (Abcam, Cambridge, UK) at 1 : 100 dilution and claudin-7, rabbit polyclonal (Abcam) at 1 : 100 dilution.

Antigen retrieval for C35, E-cadherin and claudin-7 was carried out using sodium citrate buffer (18 *μ*M citric acid, 82 *μ*M sodium citrate, pH 6.0). Antigen retrieval for Twist was carried out using Tris/EDTA buffer (1 mM EDTA, 10 mM Tris-HCl base, pH 8.0). Standard immunohistochemistry protocol was carried out using the REAL EnVision mouse/rabbit kit (Dako), according to the manufacturer's instructions. For C35, comparative staining showed that automated AQUA immunofluorescence and manual immunohistochemistry scores correlated as follows: <100 : 0; 100–200 : 1+ 201–300 : 2+ and >300 : 3+.

HER2 immunohistochemistry was carried out using HercepTest (Dako), according to the manufacturer's instructions; with antigen retrieval at 96°C for 40 min. Staining was carried out on Autostainer (Dako). HER2 assessment was carried out according to the ASCO/CAP guidelines (Wolff *et al*, 2007). HER2 tumours were defined as positive only when the immunohistological score was 3+ and *HER2* amplification was subsequently verified by FISH.

### Cell lines, transfection and foci formation

The BT474, T47D, MBA-MD-231 and SKBr3 cell lines were obtained from the American Type Culture Collection. BT474, MBA-MD-231 and SKBr3 cells were cultured in RPMI 1640 (Invitrogen, Paisley, UK) supplemented with 10% donor bovine serum, 50 U ml^−1^ penicillin and 50 mg ml^−1^ streptomycin. T47D cells were cultured in DMEM (Invitrogen) supplemented with 10% donor bovine serum, 50 U ml^−1^ penicillin and 50 mg ml^−1^ streptomycin.

H16N-2 is an immortalised cell line derived from normal breast epithelium that does not over-express C35 (a kind gift from Dr V Band; Band and Sager, 1991). H16N-2 cells were cultured in DFCI media (Evans *et al*, 2006) or commercial MEGM (Lonza, Slough, UK) supplemented with 5% serum. The culture media were supplemented with 0.5 mg ml^−1^ G418 for vector selection. For detection of foci formation, we stained confluent monolayers with crystal violet (0.1% crystal violet, 20% ethanol) for 5 min, followed by de-stain rinse with water.

### C35 and ITAM mutants through transfection

The coding region for human C35 protein was cloned into plasmid vector pIRESneo3 (Clontech, Mountain View, CA) at *Bsi*WI and *Bam*HI restriction sites. Plasmid DNA encoding wild-type (wt), Y39F/Y50F ITAM mutant or empty vector was transfected into host cells using Lipofectamine 2000 (Invitrogen) in OptiMem transfection medium following the manufacturer's protocol. Transfection medium was replaced with growth medium after 6 h. Transfectants were selected on G418, 48 h after transfection. Bulk transfected lines were cloned using cloning discs.

### C35 recombinant cells by retroviral transduction

The coding region for human C35 protein (Evans *et al*, 2006) was cloned into retroviral vector pLXSN. To make a stable retrovirus producing line, we transfected pLXSN encoding wt C35 or empty vector into PA317 cells. Viral supernatants were collected, filter sterilised (0.45 *μ*M) and titrated in the range of approximately 10^5^ PFU per ml. H16N-2 were seeded at 3- to 5 × 10^6^ cells in a T75 flask and incubated with 3 ml of viral supernatant and 2 *μ*g ml^−1^ polybrene at 37°C for 6 h. Infection media were replaced with DFCI growth media and 0.5 *μ*g ml^−1^ G418 was added at 48 h after infection. Bulk transduced lines were cloned by limiting dilution. Cell lines were assessed for C35 expression by western blot and/or immunofluorescence staining with C35 mouse monoclonal antibody (clone 1F2.4.1; Vaccinex) on fixed and permeabilised cells.

### Soft agar colony formation assays

Triplicate wells of a six-well plate were seeded with uniform H16N-2 or MDA-MB-231 cell suspension diluted in DFCI, 0.33% agar (4 × 10^3^ cells per well), which was layered over a bottom layer containing 0.625% agar. Plates were incubated up to 5 weeks at 37°C, fresh media were added to each well every week to replenish nutrients and moisture. Presence of colonies was detected under light microscope and visual inspection, at which point colonies were stained with *P*-iodonitrotetrazolium violet dye (Sigma-Aldrich, Gillingham, UK). Iodonitrotetrazolium violet stock (dissolved in 95% ethanol at 20 mg ml^−1^) was diluted to 1 mg ml^−1^ in PBS and 0.25 ml was added to each well. After overnight incubation at 37°C, visible colonies were counted in each well; counts from three wells were averaged. The number of colonies was normalised by multiplying the average number of soft agar colonies by the ratio of attached growth colonies normal to attached growth colonies transfectant. The attached growth assay was carried out at the same time as the soft agar assay, where 1/100 of each soft agar dilution was seeded into 100 mm dish. At 10–12 days after seeding, the dishes were stained with crystal violet and colonies were counted (Foos *et al*, 1998). Similar results were obtained in three independent experiments.

### Collagen invasion assays

To characterise the mode of invasion of C35-expressing cells, we carried out collagen invasion assays essentially as previously described (Amjad *et al*, 2007). These assays are different from traditional Boyden chambers in several aspects: (1) the material used is a mixture of collagen and fibroblasts, generating a lattice of stroma-like substance; (2) the presence of live fibroblasts allows for continuing interaction with the epithelial cells; (3) importantly, the cells are examined as they invade the collagen lattice, not only measuring the number that have invaded right through the material.

Briefly, rat collagen I solution was mixed with 10^5^ human breast fibroblasts (obtained from reduction mammoplasty, referenced in Amjad *et al*, 2007) per lattice and left to contract in fibroblast media (DMEM (Invitrogen) supplemented with 10% serum, 50 U ml^−1^ penicillin and 50 mg ml^−1^ streptomycin) for 4–7 days. When the lattices were of the required size (approximately four-fold contraction), 3 × 10^5^ H16N-2 cells from the desired lines were seeded on top of the lattices and incubated as submerged cultures for 3–4 days in H16N-2 media. To induce invasion, we raised the lattices to the air/liquid interface and incubated for further 7 days before the they were fixed in 10% phosphate-buffered formalin and embedded in wax.

### RNA extraction and RT–PCR

RNA was extracted by RNeasy Mini kit (Qiagen, Crawley, UK), evaluated on Agilent (South Queensferry, UK) Bioanalyzer (RIN>9.5) and labelled using Illumina TotalPrep RNA amplification kit (Applied Biosystems/Ambion, Austin, TX, USA) according to the manufacturers' instructions. Triplicate samples from whole invasion assays (1500 ng cRNA each) were hybridised to Illumina BeadChips, according to the manufacturer's instructions. Whole-genome gene expression analysis was performed using Illumina HumanRef-8 v3 Expression BeadChip and BeadArray Reader. Microarray data were analysed using packages within Bioconductor (Gentleman *et al*, 2004; http://www.bioconductor.org) implemented in the R statistical programming language (http://www.r-project.org/). The gene expression data were normalised using quantile normalisation within the bead array package (Dunning *et al*, 2007) and differential gene expression was assessed using significance analysis of microarrays (SAM; Tusher *et al*, 2001) using the siggenes package. The data set of Herschkowitz *et al* (2007) was downloaded from the UNC Microarray Database (https://www.genome.unc.edu/).

Confirmation of gene expression patterns from biological triplicates of invasion assays was carried out using the QuantiTect SYBR Green kit (Corbett/Qiagen, Crawley, UK) on a Corbett Rotor-Gene 3000. Primers for *CDH1* were: forward 5′-CGGAGAAGAGGACCAGGACT-3′, reverse 5′-GGTCAGTATCAGCCGCTTTC-3′ for *CLDN7*: forward 5′-AAAATGTACGACTCGGTGCTC-3′, reverse 5′-AGACCTGCCACGATGAAAAT; for TBP: forward 5′-GGGGAGCTGTGATGTGAAGT-3′, reverse 5′-CCAGGAAATAACTCTGGCTCA-3′ for *ACTB*: forward 5′-CCTTCCTGGGCATGGAGTCCT-3′, reverse 5′-GGAGCAATGATCTTGATCTT-3′. QuantiTect Primer Assays (Qiagen) were used for *KRT8*, *MAL2*, *TACSTD1* and *SPINT2*. PCR programme was identical for all genes: 95°C, 15 min (94°C, 15 s; 56°C, 30 s; 72°C, 30 s) × 50 cycles; 72°C, 5 min. Standard reference human cDNA was from Clontech (catalogue no. 639654), random primed, ∼50 ng RNA equivalent per *μ*l was used for quantification of mRNA expression. Final normalisation as shown in Figure 4 was performed against the geometrical mean of *ACTB* and *TBP* levels.

### Flow cytometry

shRNA constructs were cloned into Open Biosystems/ThermoFisher, Huntsville, AL) lentiviral inducible system; cell lines generated using non-silencing and shRNA-598 (agagagacactctccatgaaca) were evaluated for both C35 and Her2 expression. FACS analysis: cells were cultured in complete medium in the presence or absence of 0.5 *μ*g ml^−1^ doxcycline for at least 7 days, collected with trypsin and re-suspended in FACS buffer (PBS (pH 7.2), 1% BSA). For HER2 staining, cells were incubated with 2 *μ*g ml^−1^ biotinylated Herceptin or Remicade as human IgG1 isotype control, for 20 min on ice, followed by washing and incubation with 2 *μ*g ml^−1^ streptavidin–APC. For C35 staining, cells were fixed and permeabilised according to the manufacturer's instruction using Invitrogen Fixation and Permeabilization kit GAS-004, and stained with 0.5 *μ*g ml^−1^ C35 monoclonal antibody 1F2 or mouse IgG (BD Biosciences, catalogue no. 557732) conjugated to Alexa 647 for 45 min at room temperature. Cells were washed in FACS buffer and analysed on FACSCalibur. Samples were run in triplicate and averaged; ratio of median fluorescence intensity was plotted.

### Three-dimensional cultures

Three-dimensional cultures have been used to study the behaviour of MECs in the presence of reconstituted basement membrane (Debnath and Brugge, 2005). This assay is particularly useful in observing oncogenic potential, by measuring morphological changes of the acinar structures formed in the culture. Such changes include enlarged acinar structures, local invasion and lack of lumen formation (Debnath and Brugge, 2005). We previously studied the effects of ITAM-containing proteins using 3D cultures (Katz *et al*, 2005; Grande *et al*, 2006), accurately predicting their contribution to tumour formation *in vivo* (Ross *et al*, 2006).

Cells (5 × 10^3^ cells per chamber) were cultured on Matrigel (BD Biosciences) cushions following the precise protocol published previously (Debnath *et al*, 2003) using the usual cell culture media with the addition of 2% Matrigel. The structures were analysed, at a magnification of × 20, on a Leitz (Microscope Co., Glasgow, UK) Dialux 20 equipped with an Insight 4 video camera and SPOT software (Diagnostic Instruments, Sterling Heights, MI, USA). Quantification of structure size was carried out using a 10 × 50 *μ*m grid reticule (Fisher Scientific, ThermoFisher, Huntsville, AL, USA), with 20–50 structures counted from each chamber. The inhibitors BAY61-3606 and piceatannol (Merck, Nottingham, UK) and trastuzumab/Herceptin (Roche Diagnostics, Penzberg, Germany) were added as follows (Figures 5 and 6):

(5a) T47D cells treated for 14 days with the Syk inhibitors BAY61-3606 (100 nM) or piceatannol (1 *μ*g ml^−1^) (added twice: at days 8 and 11).

(5b) BT474 cells treated for 13 days with trastuzumab (20 *μ*g ml^−1^) and/or BAY61-3606 (50 nM) (twice: at days 7 and 10).

(6b) Y39F/Y50F ITAM mutant or wt C35-expressing cell lines were treated for 14 days with the Syk inhibitor BAY61-3606 (50 nM, twice: at days 8 and 11).

For siRNA experiments, 10^5^ cells were plated 48 h before the 3D culture. After 24 h, the cells were transfected with 100 nM non-targeted or Syk siRNA SmartPOOLs (Dharmacon, Cramlington, UK), using Lipofectamine (Invitrogen). At 24 h after the transfection cells were collected, counted and seeded on Matrigel as described above. The effectiveness of the SmartPOOLs *vs* single siRNA was measured by qPCR (all reagents from Dharmacon) after 48 h on plastic (Supplementary Figure S3).

### Statistical analysis

For comparisons of means of structure diameters, two-tailed unpaired *t*-test was used. *P*-values were as follows:

(3c) C35, null *vs* C35pool: <0.0001; C35pool *vs* C35^hi^: 0.0041. E-cadherin, C35: null *vs* C35pool: <0.0001; C35pool *vs* C35^hi^: <0.0001.

(5a) None *vs* piceatannol: 0.0343; none *vs* BAY61-3606: 0.0119.

(5b) None *vs* trastuzumab/Herceptin: not significant; none *vs* BAY61-3606: 0.0356; none *vs* trastuzumab +BAY61-3606: <0.0001; BAY61-3606 *vs* trastuzumab +BAY61-3606: 0.0005; trastuzumab *vs* trastuzumab +BAY61-3606: <0.0001.

(5d) Non-targeted siRNA *vs* C35 siRNA: <0.0001; non-targeted siRNA *vs* HER2 siRNA: 0.0329; non-targeted siRNA *vs* Syk siRNA: 0.0104.

(6a) Neo *vs* Y39F/Y50F C35: not significant; neo *vs* wt C35: 0.0053; Y39F/Y50F C35 *vs* wt C35: 0.0026.

(6b) Y39F/Y50F C35 *vs* wt C35: 0.0111; Y39F/Y50F C35, none *vs* BAY61-3606: not significant; wt C35, none *vs* BAY61-3606: 0.0111.

(6c) Neo non-targeted *vs* Syk siRNA: not significant; neo non-targeted *vs* C35 non-targeted: 0.0011; C35 non-targeted *vs* Syk: 0.0018.

## Results

### C35 protein is co-expressed with HER2 in human breast cancer cells

**Figure 1:**
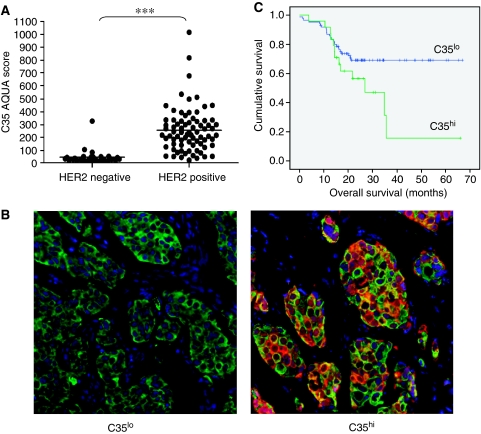
Clinical profile of C35 expression in human breast cancer. (**A**) Distribution of C35 immunofluorescence according to HER2 amplification status, as determined by fluorescence *in situ* hybridisation (^***^P<0.001). (**B**) representative examples of C35^hi^ and C35^lo^ immunofluorescence. Green: epithelial cell mask (pan-keratin); red: C35. Immunohistochemistry of C35 in primary breast cancers is shown in Supplementary Figure S1. (**C**) Kaplan–Meier survival curves according to optimal C35 cutpoint determined by minimum *P*-value method (log-rank test, *P*=0.028).

C35 protein expression was analysed by quantitative immunofluorescence using the HistoRx AQUA image analysis system (Camp *et al*, 2002) (1) to determine whether it is co-expressed with HER2 in the same cancer cells and (2) to investigate whether level of expression of protein was associated with therapeutic response to trastuzumab (Herceptin) in a retrospective clinical cohort of 122 treated patients, 32 of which were found later to be HER2 negative (Faratian *et al*, 2009). Pre-treatment C35 protein levels measured by immunofluorescence were significantly associated with *HER2* copy number amplification assessed by FISH (Figure 1A and B; mean AQUA score HER2 not amplified=47.8 (s.d. 55.2; range 17.4–327.7), mean AQUA score HER2 amplified=255.2 (s.d. 170.9; range 40.1–1014.9); Mann–Whitney *U*-test, *P*<0.0001). In cancers with no *HER2* amplification expression of C35 was uniformly low in all but two cases, with AQUA scores of less than 100.

We next sought to establish whether quantitative C35 expression was associated with response to trastuzumab as measured by the overall survival time. In univariate analysis high C35 expression (cut point AQUA score 304; minimum *P*-value method) was associated with worse overall survival (Figure 1C; log-rank test *P*=0.0285), along with stage, ER status and chemotherapy regimen (Supplementary Table S1). However, only stage was associated with overall survival in a Cox regression multivariate analysis. Analysis of C35 expression in *HER2*-amplified tumours similarly did not yield a significant association with survival (Figure 1C).

### Over-expression of C35 leads to EMT-mediated cell invasion

**Figure 2:**
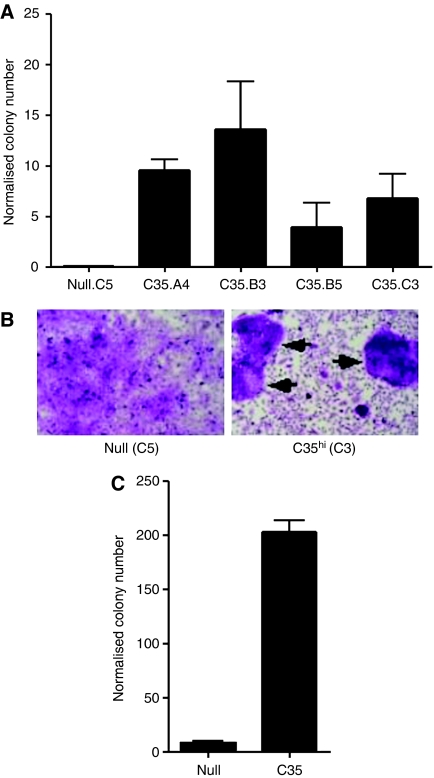
C35 expression in mammary epithelial cells leads to colony formation in soft agar. Quantification of soft agar assays in (**A**) H16N-2 cells expressing empty retroviral construct (null clone) or C35 (clones A4, B3, B5 and C3) and in (**C**) MDA-MB-231 cells (null or C35 transfectant pools). (**B**) Foci formation (marked by arrows) assay results are shown for null.C5 and the C35.C3 (high expressing) clones.

We carried out colony formation assays in soft agar to test whether C35 can induce MEC transformation. For this purpose, the normal MEC line H16N-2, which has been used previously for cell transformation assays (Burwell *et al*, 2007; Rhodes *et al*, 2009), was retrovirally transduced with wt C35. Colonies expressing high levels of C35 consistently formed enlarged structures in soft agar, whereas empty vector-expressing controls did not (Figure 2A). In contrast to the C35 transfectant pool, two of H16N-2 clones expressing high levels of wt C35 protein also showed foci formation when grown on plastic (Figure 2B; data not shown). In the breast cancer cell line MDA-MB-231, which normally expresses very low levels of C35, similar to those in the H16N-2 parental line (Evans *et al*, 2006), C35 expression was able to transform the MDA-MB-231 cell line at levels exceeding the transforming potential observed in the H16N-2 cell line (Figure 2C).

**Figure 3:**
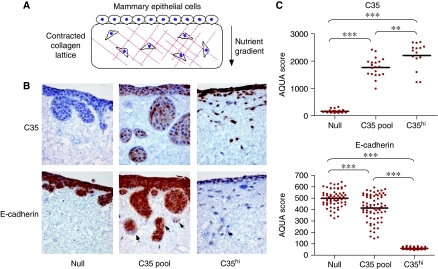
C35-expression leads to invasive phenotype, associated with epithelial to mesenchymal transition. (**A**) Schematic illustration of the invasion assay set-up in a collagen gel containing fibroblasts overlaid with epithelial cells. (**B**) H16N-2 cells expressing empty vector (null, left panels), C35-expressing pool (middle panels) and C35hi-expressing cells were stained by immunohistochemistry for C35 and E-cadherin. Note specific areas of E-cadherin loss in C35 pool (arrows). (**C**) Quantification of C35 and E-cadherin by AQUA is shown. Bar indicates mean of measurements (*P*-value indicators: ^*^<0.05; ^**^<0.01; ^***^<0.001).

We previously reported that ITAM-containing proteins such as MMTV Env can induce an invasive phenotype in human MECs (Katz *et al*, 2005), likely to be caused by an EMT (Katz *et al*, 2005; Grande *et al*, 2006). It has also been shown that C35 promotes migration and invasion in prostate cancer cell lines (Dasgupta *et al*, 2009). To determine whether C35 expression also results in a similar behaviour in MEC, we used an invasion assay that used collagen lattices closely resembling breast stroma *in vivo* (Amjad *et al*, 2007; data not shown). The stroma-like lattices were generated by rat collagen I, contracted by seeding breast fibroblasts into the collagen gel. After the lattices contracted, MECs were seeded on top and invasion was induced by a nutrient gradient (Figure 3A). Although vector only (null) cells did not significantly invade the lattice, expression of C35 induced invasion. The C35 transfectant pool, which had variable levels of C35 expression, invaded mostly in large clusters of cells (Figure 3B). Three high-expressing clones showed complete transition to spindle cell phenotype, with single cells invading deep into the lattice (Figure 3B; Supplementary Figure S2). We chose one high-expressing clone, C35.C3, for further molecular characterisation (Figure 3C). Gradual loss of E-cadherin was apparent, occasionally in the C35-expressing pool and entirely within the C35^hi^ clone (Figure 3B and C). Finally, all three major transcription factors known to be involved in EMT were examined. Slug expression was not detected and the level of Snail expression did not change in any C35-expressing cells. In contrast, Twist protein expression correlated positively with C35 expression (data not shown).

We carried out whole-genome expression array analysis to examine which transcripts correlate with C35 expression in the collagen invasion assays (raw gene expression files are publicly available from the caBIG-supported Edinburgh Clinical Research Facility Data Repository: https://www.catissuesuite.ecmc.ed.ac.uk/caarray/). Of the top 100 ranked differentially expressed genes by SAM (Tusher *et al*, 2001), the majority of the genes were down-regulated (62 of 98 probes, 63%, excluding a duplicate and a discontinued probe). First, we examined using KEGG analysis pathways activated or deactivated by C35 expression. The KEGG pathway that was most significantly over-represented by the most consistently differentially expressed genes by SAM analysis was cell communication (*P*=4.33E−08, FDR=5.43E−05). The genes responsible were *KRT15*, *GJB2*, *COL17A1*, *DSG3*, *KRT13*, *KRT6A*, *KRT6B*, *KRT14*, *KRT16*, *KRT8* and *LAMA3*. Using the DAVID Bioinformatics database (Huang *et al*, 2009), we found that the processes highlighted by this pathway are cell–cell contact (adherens junctions, tight junctions, desmosomes) and ECM–receptor interactions, including focal adhesions. Interestingly, gene expression of *PLAU* (uPA), *MMP9*, *VEGFA* and *VEGFB* did not correlate with C35 levels. This suggests involvement of a different set of activated signalling pathways in MECs compared with prostate cancer cells (Dasgupta *et al*, 2009).

**Figure 4:**
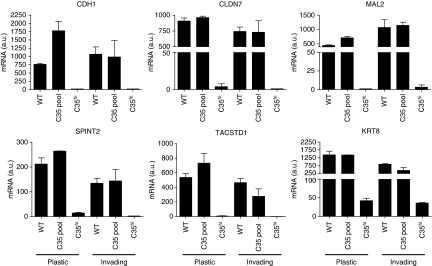
Genes down-regulated in C35-induced transformed phenotype. C35-induced down-regulation of *CDH1*, *CLDN7*, *KRT8*, *MAL2*, *TACSTD1* and *SPINT2* was observed in cells grown on plastic and in the invasion assays. Biological triplicate mRNA expression data are shown for empty vector, C35 expressing pool and C35^hi^-expressing clone (C35.C3).

When the most consistently differentially expressed genes were compared with those identified in two molecular subtypes of breast cancers linked recently to EMT, claudin low (Herschkowitz *et al*, 2007) and metaplastic breast cancers (Hennessy *et al*, 2009), a number of interesting results were discovered. Of the 23 commonly changed genes, 5 (22%) were among most changed by C35 expression: E-cadherin (*CDH1*), claudin-7 (*CLDN7*), MAL2, EpCAM (*TACSTD1*) and HAI-2 (*SPINT2*). Validation by quantitative PCR confirmed that all five genes were down-regulated by high expression of C35 in the invasion assays (Figure 4). Cells expressing high levels of C35 also down-regulated eight cytokeratin genes (out of 98 top ranked, 8%), also consistent with loss of epithelial phenotype. These included keratin-8 (*KRT8*, Figure 4), which is often down-regulated in EMT-like breast tumours (Herschkowitz *et al*, 2007; Hennessy *et al*, 2009). Both loss of cell–cell contact and down-regulation of cytokeratins have been linked with EMT and are thought to enable cancer cell invasion (Levayer and Lecuit, 2008).

### C35-induced cell transformation is dependent on the function of its ITAM

Previous studies have used 3D cell culture, in which MECs are grown on reconstituted basement membrane (Matrigel) and form spherical structures resembling the terminal ductal lobular units in the breast. These cell cultures show many *in vivo* properties of MECs. This model has been extensively used to study oncogenic phenotypes (Debnath and Brugge, 2005). C35 contains an ITAM, a motif found in glycoproteins of oncogenic retroviruses, that is linked to epithelial cell transformation through the protein tyrosine kinase Syk (Katz *et al*, 2005; Lu *et al*, 2006; Wang *et al*, 2006). Syk binds to the ITAM through its tandem SH2 domains and activates multiple growth signalling pathways, including PI3K, PLC*γ*, Ras/MAPK and NF*κ*B, among others (Underhill and Goodridge, 2007).

**Figure 5:**
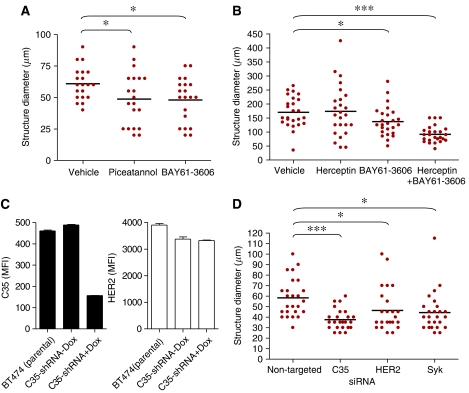
Inhibition of C35 and Syk reduces mammary epithelial cells acinar structure size. (**A**) Quantification of 3D structure size in T47D cells at day 14 after treatment with the Syk inhibitors BAY61-3606 or piceatannol. (**B**) Quantification of structure size in BT474 cells at day 13 after treatment with Herceptin (trastuzumab) and/or BAY61-3606. (**C**) Knockdown of C35 by siRNA in BT474 cells (left panel) has no effect on HER2 surface expression (right panel) as determined by flow cytometry. (**D**) Quantification of structure size of BT474 cells treated with non-targeted, C35, HER2 or Syk siRNA, at day 6 of 3D culture.

**Figure 6:**
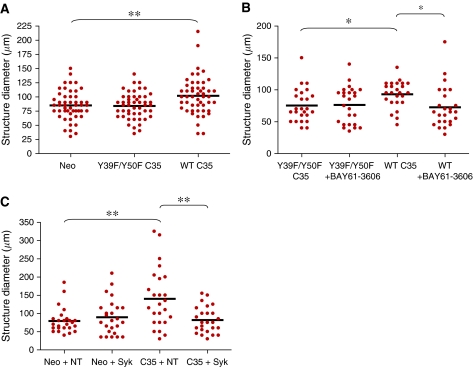
C35 expression in normal mammary epithelial cells leads to cell transformation in 3D cultures. (**A**) H16N-2 acinar structures (at day 11) expressing empty vector (Neo), Y39F/Y50F ITAM mutant or wild-type (wt) C35 protein. (**B**) Quantification of structure diameter in Y39F/Y50F ITAM mutant or wt C35-expressing cell lines at day 14 after treatment with the Syk inhibitor BAY61-3606 (50 nM, twice: at days 8 and 11). (**C**) Quantification of structure size of Neo and wt C35-expressing H16N-2 cells treated with non-targeted, Syk siRNA, at day 6 of 3D culture.

We determined the C35 and HER2 status of three breast cancer cell lines, as well as Syk expression. BT474 and SKBr3 lines harbour *HER2* and *C35* gene amplification and show high levels of mRNA expression of these genes (Supplementary Figure S4). T47D cells have no *HER2* gene amplification and they express moderate levels of C35 (21-fold less than SKBr3 cells, 4-fold more than MCF10A cells). T47D cells are sensitive to Syk inhibition, by piceatannol or BAY61-3606 treatment (Yamamoto *et al*, 2003; Figure 5A), similar to that of the H16N-2 C35-expressing line in 3D culture (Figure 6B). Therefore, the response of *HER2*-amplified cells to Syk inhibition was determined. BT474 cells were chosen as they form non-polarised but well-defined ‘mass’ 3D structures (Kenny *et al*, 2007), similar to those generated by T47D cells. Treatment with Syk inhibitors, or Syk siRNA, reduced the size of BT474 3D structures (Figure 5D). This effect was unlikely due to changes in HER2 expression (Figure 5C). Syk inhibition combined with Herceptin (trastuzumab) resulted in even smaller structures, similar in size to those seen with immortalised, but non-transformed, cell lines (Figure 5B).

We generated H16N-2 cell transfectant pools expressing the wt C35 protein or its Y39F/Y50F ITAM mutant. When grown in reconstituted basement membrane (3D culture), MECs expressing ITAM-containing proteins showed a transformed phenotype. This phenotype included enlargement of the acinar structures and was dependent on functional ITAM in these proteins (Katz *et al*, 2005; Grande *et al*, 2006). Consistent with these previous observations, when cultured in 3D, C35-expressing cells formed enlarged structures in comparison to empty vector-expressing cells (*t*-test, *P*=0.0053). Immunoreceptor tyrosine-based activation motif mutant C35-expressing cells formed similar structures to those of vector-expressing cells (Figure 6A).

Growth of C35-expressing H16N-2 cells was sensitive to Syk inhibition in 3D culture (Figure 6B) similar to other cell lines expressing ITAM-containing proteins (Katz *et al*, 2005; Grande *et al*, 2006). This was confirmed by siRNA knockdown for both Syk and C35 (Figure 6C). We also observed down-regulation of Syk mRNA in H16N-2 expressing the Y39F/Y50F ITAM mutant, compared with those expressing wt C35 (data not shown). This observation supports the view that Syk interacts with functional ITAM-containing C35.

## Discussion

HER2/ErbB2 amplification is a frequent and well-studied event in breast and other cancers. The genetic fragment being amplified is commonly known as the *HER2* amplicon. The smallest region of amplification of the *HER2*/*ERBB2* amplicon on human chromosome 17q12 contains 14 core genes, of which *STARD3*, *TCAP*, *PNMT*, *PERLD1*, *ERBB2*, *GRB7*, *GSDML* and *C17orf37*/C35 are over-expressed when amplified (Kauraniemi and Kallioniemi, 2006; Marchio *et al*, 2008). The function of HER2, in breast cancer in particular, has been subject to intense research efforts, culminating in the design of both small molecule inhibitors and monoclonal antibodies in treatment of HER2^+^ patients (Bublil and Yarden, 2007). Recent efforts have concentrated on understanding how co-amplification of *HER2* with the non-core amplicon gene Topoisomerase II (*TOP2A*) may affect response to chemotherapy (Pritchard *et al*, 2008). Much less is known about the functional importance of the core genes co-amplified with HER2. One of the best studied of these core genes is *GRB7*. Co-expression of Grb7 and HER2 facilitates HER2 signal transduction and functions synergistically for tumour formation (Stein *et al*, 1994; Bai and Luoh, 2008). Tumours co-expressing high levels of Grb7 and HER2 have a worse outcome than those with only higher levels of HER2 (Nadler *et al*, 2010), in line with the clinical data presented here. Both *GRB7* and another core gene, *STARD3*, contribute to the growth of HER2-amplified cell lines *in vitro* (Kao and Pollack, 2006).

Here, we show that primary breast cancers have high levels of C35 protein expression when harbouring *HER2* gene amplification, and that over-expression of C35 and HER2 protein is correlated in both breast cancer cell lines and primary tumours, in agreement with previous findings (Evans *et al*, 2006). It is estimated that tumours can express 70–100 times the normal breast tissue C35 transcript level (Evans *et al*, 2006). Cell lines expressing high levels of C35 showed high invasive behaviour *in vitro*. The overall phenotype is consistent with EMT, including down-regulation of E-cadherin and up-regulation of Twist. Interestingly, more gene transcripts were down-regulated than up-regulated among the 100 most changed transcripts. This raises the possibility of common suppression mechanism of transcription, downstream of C35 expression. A study in a pancreatic cancer cell line has suggested that the protein inhibitor specific for HGF activator-1 (HAI-1), an HAI-2 homologue, may activate an EMT programme in these cells by up-regulating the transcription factor SIP-1/ZEB-2 and consequently repressing E-cadherin (Cheng *et al*, 2009).

We found that tyrosine mutation in the ITAM of C35, or Syk kinase inhibition, is sufficient to abolish the potential of C35 protein to cause enlargement of acinar structures in 3D cell culture. Studies in DLBCL lines have shown that some, but not all tumours, expressing ITAM-containing proteins may respond to Syk inhibition (Chen *et al*, 2008). Evidence in this study using C35-expressing MEC lines has supported this strategy *in vitro*. Syk expression and activation are also modulated by extracellular matrix, through integrin signalling (Zhang *et al*, 2009). Syk promotes cell–cell contact on plastic (Zhang *et al*, 2009) and its genetic knock-down promotes cell mobility and invasion (Sung *et al*, 2009; Zhang *et al*, 2009). Syk may also have a tumour suppressor function in breast cancer through its kinase activity in the nucleus (Coopman *et al*, 2000; Sung *et al*, 2009). A plausible mechanism is that the interaction of C35 with Syk mimics global knock-down of Syk by changing its localisation away from integrins (Zhang *et al*, 2009) or the nucleus (Coopman *et al*, 2000). When activated in the cytoplasm, Syk functions as a promoter of cell growth (Zhou and Geahlen, 2009), consistent with the function postulated in this study. Our study results indicate that recently described Syk inhibitors (Braselmann *et al*, 2006; Chen *et al*, 2008) may be effective in C35 over-expressing breast cancer cells and thus have therapeutic value.

Other therapeutic approaches may be developed to take advantage of these findings in the treatment of human breast cancer, including the development of inhibitors of C35 interaction with proteins other than Syk, such as the novel ITAM-interacting protein Shb (Matskova *et al*, 2007). The Src kinase Lyn is unlikely to be involved in C35-induced EMT, because Lyn mRNA levels are reduced by approximately five-fold in C35^hi^ cells, in comparison with both null and C35 transfectant pool cells.

In conclusion, we show here that the *HER2* amplicon contains a second oncogene, C35, in the context of breast cancer. Our observations suggest that targeting C35 as well as HER2 may be beneficial for patients with *HER2*-amplified breast cancers. C35/C17orf37 has recently been included in an expression signature predicting metastatic risk in node-negative breast cancer after chemotherapy (Jezequel *et al*, 2009). This signature does not include HER2, therefore suggesting a possible autonomous role for C35, and warrants further investigation.
