# Supplementary figures and images for: A gene on the HER2 amplicon, C35, is an oncogene in breast cancer whose actions are prevented by inhibition of Syk
Source: Br J Cancer. 2010 Jul 13;103(3):401–10. doi: 10.1038/sj.bjc.6605763 (PMC2920017; doi:10.1038/sj.bjc.6605763)

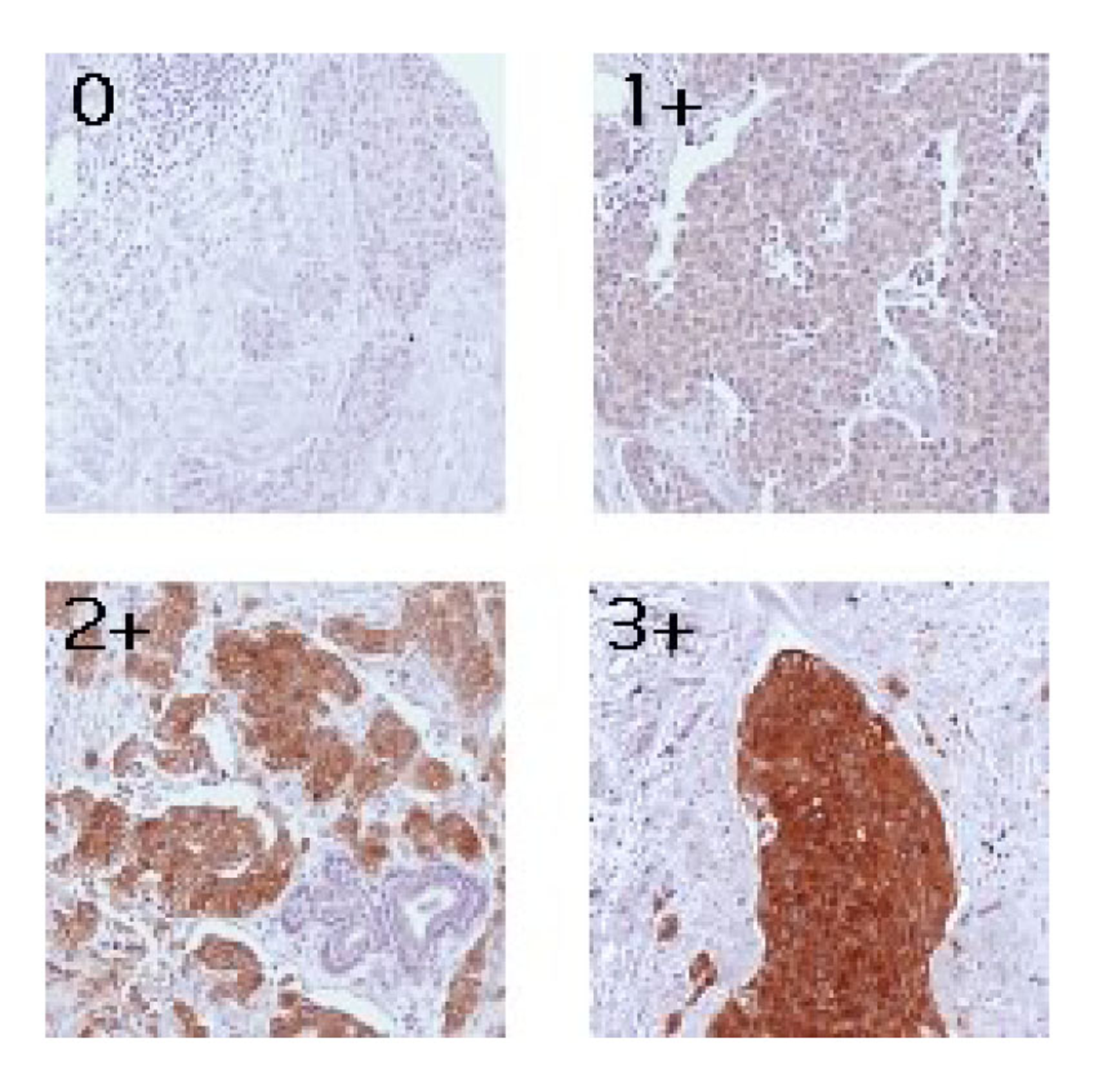

Supplement: Supplementary Figure 1 [file 6605763x1.tif]

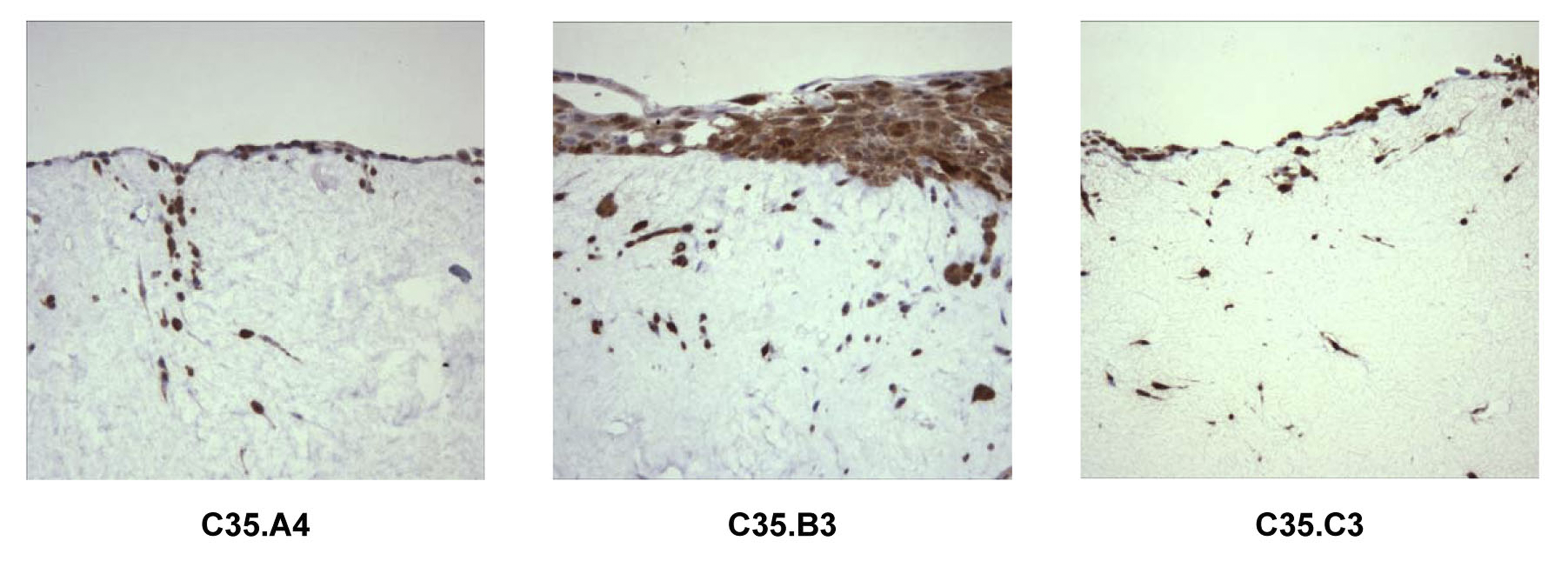

Supplement: Supplementary Figure 2 [file 6605763x2.tif]
