# Supplementary material for: A gene on the HER2 amplicon, C35, is an oncogene in breast cancer whose actions are prevented by inhibition of Syk
Source: Br J Cancer. 2010 Jul 13;103(3):401–10. doi: 10.1038/sj.bjc.6605763 (PMC2920017; doi:10.1038/sj.bjc.6605763)
Supplement: Supplementary Information [file 6605763x5.doc]

Supplementary Table 1: Summary of patient characteristics (for figure 1): age, prognostic index, grade, stage, node, ER and HER2 status and chemotherapy given.

Supplementary Figure 1: Immunohistochemistry staining for C35. Protein expression is scored as negative (0), weak (1+), intermediate (2+) or high (3+).

Supplementary Figure 2: Immunohistochemistry staining for C35 in H16N-2 C35^hi^ clones cultured in the invasion assay set-up. Note the spindle-like cell morphology in all clones.

Supplementary Figure 3: mRNA expression levels of C35, Syk and HER2 in BT474 after siRNA transfection.

Supplementary Figure 4: mRNA expression levels of C35 and HER2 in selected human mammary epithelial lines showing high levels of expression in BT474 and SKBr3 cells relatively to normal MEC line, MCF10A.

Supplementary Table 1

| ***Cohort variable*** | ***Number*** | ***Percentage*** | **Log-rank p-value** |
| --- | --- | --- | --- |
| **Age, years** |  |  | 0.46 |
| <50 | 49 | 40.1 |  |
| >50 | 73 | 59.9 |  |
| NK | 0 | 0 |  |
| **Prognostic Index** |  |  | 0.22 |
| <3.4 | 2 | 1.6 |  |
| 3.4-5.4 | 47 | 38.5 |  |
| >5.4 | 62 | 50.8 |  |
| NK | 11 | 9.0 |  |
| **Grade** |  |  | 0.80 |
| 1 | 1 | 0.8 |  |
| 2 | 19 | 15.6 |  |
| 3 | 99 | 81.1 |  |
| NK | 2 | 1.6 |  |
| **Tumour Stage** |  |  | 0.024 |
| 1 | 35 | 28.7 |  |
| 2 | 64 | 52.5 |  |
| 3 | 12 | 9.8 |  |
| 4 | 3 | 2.5 |  |
| NK | 8 | 6.6 |  |
| **Node stage at diagnosis** |  |  | 0.20 |
| Negative | 26 | 21.3 |  |
| Positive | 87 | 71.3 |  |
| NK | 9 | 7.4 |  |
| **ER status** |  |  | 0.038 |
| >3 | 72 | 59.0 |  |
| ≤3 | 41 | 33.6 |  |
| NK | 9 | 7.3 |  |
| **HER2 status** |  |  | 0.38 |
| Positive | 90 | 73.7 |  |
| Negative | 32 | 26.3 |  |
| NK | 0 | 0 |  |
| **Chemotherapy** |  |  | <0.0001 |
| Anthracycline-containing | 66 | 54.1 |  |
| Taxane-containing | 53 | 43.4 |  |
| NK | 3 | 2.5 |  |
